# Understanding students’ readiness for interprofessional learning in an Asian context: a mixed-methods study

**DOI:** 10.1186/s12909-016-0704-3

**Published:** 2016-07-15

**Authors:** Endang Lestari, Renée E. Stalmeijer, Doni Widyandana, Albert Scherpbier

**Affiliations:** Medical Education Unit, Faculty of Medicine, Universitas Islam Sultan Agung, Semarang, Indonesia; School of Health Professions Education, Faculty of Health, Medicine and Life Sciences, Maastricht University, Maastricht, The Netherlands; Department of Medical Education, Faculty of Medicine, Gadjah Mada University, Yogyakarta, Indonesia

**Keywords:** Interprofessional education (IPE), Readiness for Interprofessional Learning Scale (RIPLS), Interprofessional collaboration, Role blurring

## Abstract

**Background:**

Healthcare is generally provided by various health professionals acting together. Unfortunately, poor communication and collaboration within such healthcare teams often prevent its members from actively engaging in collaborative decision-making. Interprofessional education (IPE) which prepares health professionals for their collaborative role in the healthcare system may partially address this problem. This study aimed to investigate: 1) students’ readiness for IPE in an Asian context, 2) the most important factors influencing students’ perceptions of IPE, 3) the reasons underlying such perceptions, and 4) the factors mitigating or promoting their sense of readiness.

**Methods:**

To identify students’ perceptions of IPE, we administered the Readiness for Interprofessional Learning Scale (RIPLS) to 398 in approximately 470 students from a range of health professions (medicine, nursing, midwifery and dentistry). The questionnaire included factors that could potentially influence readiness for IPE as found in the literature (GPA, etc.). To enhance our understanding of the responses to the RIPLS and to explore the reasons underlying them, we conducted 4 mono-professional focus group discussions (FGDs). We ran a statistical analysis on the quantitative data, while performing a thematic content analysis of the qualitative data using ATLAS.ti (version 7).

**Results:**

Medical students seemed to be the most prepared for IPE. Students’ perceptions of IPE were conditioned by the study programme they took, their GPA, intrinsic motivation and engagement in the student council connoting experience of working with students from different programmes. Focus groups further revealed that: 1) early exposure to clinical practice triggered both positive and negative perceptions of IPE and of its importance to learning communication and leadership skills, 2) medical students caused insecurity and disengagement in other students, 3) medical students felt pressured to be leaders, and 4) there was a need to clarify and understand each other’s profession and the boundaries of one’s own profession.

**Conclusion:**

Students were generally favourable to IPE, appreciating the opportunity it offered them to hone their interprofessional leadership, collaboration and communication skills and to learn to address the problem of role blurring. Hence, we judge the Asian context ready to implement IPE, allowing health professions students in Asian countries to reap its benefits. The present study revealed several important reasons underlying students’ positive and negative perceptions of IPE implementation which may be addressed during the interprofessional learning process.

**Electronic supplementary material:**

The online version of this article (doi:10.1186/s12909-016-0704-3) contains supplementary material, which is available to authorized users.

## Background

The complex nature of today’s healthcare, which not only aims to cure and prevent disease but also to promote health, requires effective collaboration between various healthcare professionals. However, interprofessional collaboration is not self-evident and is fraught with problems such as ineffective communication, poor interprofessional relationships, a lack of trust between team members, and an underestimation of other health professionals’ roles [1]. These factors hinder the effective involvement of all team members in collaborative decision-making regarding patient care and the implementation of healthcare services.

To partially address this problem, the WHO has recommended the introduction of interprofessional education (IPE) which helps future healthcare professionals prepare for their collaborative role in the healthcare system. IPE offers students from different health professions the opportunity to learn with, from and about each other’s profession and has been recognised as a means to safely promote and develop the collaboration skills students require in their later profession. Research has revealed that health professionals who were trained to collaborate as a team in an interprofessional educational setting during their student years were far more likely to be effective collaborators in their future professional clinical setting [2]. Although the implementation of IPE has been studied in a number of settings [3, 4] its implementation and application in the Asian region has received scant attention [5]. Like other peoples in the world, the Asian population is facing highly complex health problems that require interprofessional collaboration. In addition, the Asian region has a very strong culture of social hierarchy [6, 7], which translates into a large power distance between its people, also between doctors and nurses. Doctors are considered to hold the highest positions in society, whereas other health professionals such as nurses and midwives are marginalised. This situation further complicates effective interprofessional collaboration within healthcare teams and could potentially undermine successful implementation of IPE in higher education. Compounding matters in most Southeast Asian countries is that the boundaries between healthcare roles are frequently blurred and that the education system for healthcare professionals lacks standardisation [8]. From an educational management point of view, one could indeed anticipate that differences in timetables, in student numbers across the various health professions departments, in curricula and teaching approaches, and in assessment strategies pose a problem to effective IPE implementation [9, 10]. Even more challenging than this, however, are considered to be students’ attitudes towards the new learning approach to be implemented, in this case IPE [11, 12]. It has also been demonstrated that students’ attitudes towards and their perceptions of an educational approach are culture-bound [13]. Theoretically speaking, the hierarchical nature of Asian cultures clashes with the inherent IPE principle that all health professionals are equal. This may indicate that Asian students will be less favourable to IPE than, perhaps, students from certain Western cultures that are less hierarchical. Yet, to our knowledge, there have been few studies [5, 14, 15] that addressed students’ attitudes towards IPE in an Asian context.

The present study therefore seeks to answer the following research questions:

1. Are students in an Asian context ready for IPE?
2. What are the most important factors influencing students’ perceptions of IPE?
3. How do students explain their readiness for IPE?
4. Which factors do they describe that either mitigate or promote their sense of readiness for IPE?

## Methods

### Context

In Indonesia all undergraduate health professions programmes have introduced interprofessional collaboration skills into their core curricula. However, very few of these universities have actually incorporated an IPE programme facilitating collaborative learning by multidisciplinary student teams into their curriculum. Universitas Islam Sultan Agung is one such university that has not yet implemented IPE, but it has the intention to develop an IPE curriculum for its medical, nursing, midwifery and dentistry programmes. For this purpose, we conducted a survey of students’ perceptions of IPE. The named programmes differ in length and in the duration of their pre-clinical and clinical phases. While the medical, nursing and dentistry programmes all have 5-year curricula, the midwifery programme spans 3.5 years. Their clinical phases each start after 3.5, 4, 4 and 3 pre-clinical years, respectively. Only midwifery and nursing students have early clinical exposure in the 2nd and 3rd year, respectively, in the form of at least 2 months of practice in a hospital or public health centre. Medical and dentistry students do not gain any practical experience in their pre-clinical years other than practice in skill labs with simulated patients and manikins. Learning in all programmes is mono-professional, meaning that students rarely collaborate with students from other healthcare disciplines, not even during clinical rotations. For the present study we invited medical, nursing, midwifery and dentistry students who were in their final pre-clinical year to participate.

### Research design

We selected an explanatory, sequential mixed-methods design to answer the research questions [16]. We first collected quantitative data by administering a previously validated Readiness for Interprofessional Learning Scale (RIPLS) questionnaire to healthcare students. We specifically targeted students from the medical, nursing, midwifery and dentistry programmes who were in the final year of their preclinical programme. The results of the questionnaire were then used as input for the qualitative data collection consisting of mono-professional focus group discussions aimed to understand the underlying reasons for students’ perceptions of IPE.

### Quantitative data collection: RIPLS questionnaires

To determine readiness for IPE, Parsell and Bligh [11] developed the three-dimensional Readiness of healthcare students for Interprofessional Learning Scale (RIPLS) questionnaire, consisting of 19 items. This instrument explores attitudinal factors that are important to consider when designing IPE, such as respect for one’s own and other’s professional identity, knowledge and roles. The first dimension explores whether the learner recognises the benefit of teamwork and collaboration, as well as of content and methods that teach them to work interprofessionally. The second dimension investigates positive and negative aspects of professional identity, whereas the third dimension explores perceptions of health professions’ roles and responsibilities. The instrument has a 5-point Likert scale ranging from one (strongly disagree) to five (strongly agree) with some reverse-scored items. High scores on the RIPLS indicate good readiness for interprofessional learning. The RIPLS questionnaire was translated by means of a double back translation procedure to assess the consistency between original and translated version of RIPLS. This means that an English-Indonesian translator first translated the English version of the questionnaire into Bahasa Indonesia, after which another translator translated this translation back into English. We also added a set of questions referring to factors that we knew from the literature had the potential to influence readiness for IPE. These factors were : a) study programme [17], b) respondents’ GPA [18], c) past experience of working with students from other study programmes in student associations [19], and d) motivation to study in a health professions programme [20]. Two members of the research team collected the questionnaires after lectures. Before students completed the questionnaires, the researchers explained to them what the study sought to accomplish, what type of information the questionnaires would provide, that their participation was entirely voluntary, and that their answers to the questionnaires would not affect the grades in any courses they were taking.

### Qualitative data collection: focus group discussions

To gain a better understanding of the answers provided in the questionnaires, we organised four mono-professional focus groups (FGs). We deliberately chose not to mix students from different programmes to overcome potential barriers to communication and to encourage participants in the discussion. FG participants were randomly selected on the basis of their RIPL scores: each group included about four to five students who were favourable to IPE and four to five students who were less favourable to the concept. If a student did not wish to participate, we invited a different student with similar IPE scores. All FGs were video recorded. A lecturer in community medicine (SY) who understood the concept and aims of the study facilitated the FGs with the aid of a discussion guide [21]. This guide contained the following questions for students, depending on the case: (a) why they were or were not favourable to IPE, (b) why their scores for certain items on the questionnaire were low/high, (c) whether they agreed with the plan to implement IPE in their school, and why, and (d) whether they had any suggestions for successful implementation of IPE.

### Analysis

#### Questionnaire

At the time of the study, the Indonesian translation of the RIPLS questionnaire had not been validated. We performed a factor analysis to explore its construct validity and computed Cronbach’s alpha to determine internal consistency. The suitability of the correlation matrix was determined by the Kaiser-Meyer-Olkin (KMO) measure of sampling adequacy and Bartlett’s test of sphericity. The numbers of factors retained for the initial solutions and entered into the rotation were determined with the application of Kaiser’s criterion (eigenvalues > 1). The initial factor extraction was performed using principal component analysis. Finally, we performed an exploratory factor analysis using promax rotation to define the clearer structure (see Additional file 1). The KMO index was 0.928, indicating sampling adequacy, while the Bartlett sphericity chi-square index was 5388.09, with *p* < 0.001 indicating that null hypothesis that the correlation matrix was an identity matrix and therefore unsuitable for factor analysis was rejected. Exploratory factor analysis (see Additional file 2) yielded the subscales ‘teamwork and collaboration’, ‘professional identity and role understanding’, and a third subscale consisting of one item only (Q19), which read: ‘I have to acquire much more knowledge and skills than other students/professionals in my own faculty/organisation’. Because Cronbach’s alpha cannot be determined for a one-item scale, we excluded this question from the questionnaire, so only the first two subscales remained. A plausible explanation for the low loadings on this particular item may be that it may have been interpreted to mean self-efficacy and may have inadvertently connoted a sense of superiority to others. This is contrary to Indonesian values, which stress equanimity or composure [22]. The validity test revealed that the two subscales ‘teamwork and collaboration’ and ‘professional identity and role understanding’ had Cronbach alphas of 0.944 and 0.92, respectively.

Based on the valid RIPLS, we consequently performed a Kruskal-Wallis non-parametric test to evaluate differences in RIPLS scores between the four study groups and, since scores were not normally distributed (*p* < 0.05 for all subscales), carried out a Mann-Whitney U test to analyse differences within pairs of study groups using SPSS (version 16.0). We obtained the α level of significance by using Bonferroni correction resulting in an adjusted α level of 0.008 (0.05/6).

### Focus groups

All FGDs were transcribed verbatim by two medical education experts, the results of which were summarised and sent to participants as part of a member check procedure [21]. The verbatim transcripts were made in Indonesian and coded for content, without eroding their original substance. Two medical education experts, EL and DAR, who were also native Indonesian speakers, performed the analysis. The two independently evaluated the transcripts, first by open coding, then they developed the coding categories, which they finally applied to the data. Both agreed to group students’ perceptions into subthemes of positive and negative perceptions of IPE, before starting to look for overarching themes. After this process, all members of the research team discussed findings until they reached consensus. For the thematic content analysis [21] we used qualitative data analysis and research software ATLAS.ti (version 7).

## Results

### Quantitative results: RIPLS

During the day of the study, 428 in a total of 470 students (240 medical, 120 nursing, 60 midwifery and 50 dentistry students) attended class and filled in the questionnaire. Thirty students failed to complete all parts of the questionnaire and were therefore excluded. Hence, we derived our data from a pool of 398 subjects denoting a response rate of 84.7 %. Two hundred and sixty-six female students (67 %) participated in the study, which is twice as many as their male counterparts. For 286 (71.9 %) of all respondents, the decision to apply to a health professions programme was based on personal motivation, while a mere 19.3 % had already worked with students from other study programmes in the student council. These and other demographic data are presented in Table 1.

**Table 1:** Demographics characteristics of respondents

	Midwifery		Nurse		Dentistry		Medical	
	N	%	N	%	N	%	N	%
Gender								
Male	0	0	41	41.4	11	25	80	38.1
Female	47	100	58	58.6	33	75	128	61.5
Admission								
Invitation	0		2	2	4	9.1	68	32.7
Regular test	46	97.9	73	73.7	25	56.8	116	55.8
Scholarship	1	2.1	24	24.2	15	34.1	24	11.5
Decision to study at the programme								
Own preference	23	48.9	51	51.5	32	72.7	180	86.5
Encouraged by parents	24	51.1	48	48.5	12	27.3	28	13.5
Experiencing of working with students from different programmes								
Yes	5	10.6	15	15.2	5	11.4	52	25
No	42	89.4	84	84.8	39	88.6	156	75
	Mean	SD	Mean	SD	Mean	SD	Mean	SD
Age	20.17	0.73	20.14	0.7	19.86	1.11	20.07	0.73
GPA (max score 4)	3.03	0.36	2.85	0.32	3.1	0.26	2.96	0.5

The study programme chosen, GPA, motivation to apply to a health professions programme and experience of working with students from other study programmes in a student council were factors that significantly influenced the total RIPLS score, with *p*-values of 0.000, 0.003, 0.000 and 0.008, respectively (see Table 2).

**Table 2:** Mean of each subscale based on some factors

	Teamwork and collaboration		Professional identity and role understanding		Total RIPLS score	
	Mean	*P*	Mean	*P*	Mean	*P*
Gender						
Male	55.35 ± 7.2	0.648	15.58 ± 5.5	0.908	70.92 ± 9.24	0.683
Female	55.64 ± 6.8		15.43 ± 5.8		71.08 ± 9.8	
Age						
< 21 year	55.43 ± 7.2	0.930	15.60 ± 5.8	0.700	71.03 ± 9.8	0.891
21-24 year	56.04 ± 5.93		14.97 ± 5.3		71.01 ± 9.17	
> 24 year	55.5 ± 4.94		15.5 ± 6.4		71.00 ± 1.4	
Programme						
Midwifery	54.4 ± 4.1	0.015^a^	10.9 ± 2.2	0.000^a^	65.3 ± 3,7	0.000^a^
Nursing	54.7 ± 6.2		9.9 ± 3.2		64.7 ± 5,4	
Dentistry	54.9 ± 4.5		13.1 ± 3.1		68.1 ± 5,2	
Medicine	56 ± 8.1		19.6 ± 4.1		76 ± 10	
GPA (range 0-4)						
< 2.75	53.7 ± 5.5	0.003^a^	15.2 ± 5.2	0.086	68.8 ± 8.3	0.003^a^
2.75 – 3	55.7 ± 6.2		14.6 ± 5.4		70.3 ± 8.6	
> 3	56.5 ± 7.8		16.1 ± 6.0		72.6 ± 10.1	
Motivation to study in health professions programme						
Own preference	56.1 ± 7.4	0.000^b^	16.5 ± 5.9	0.086	72.6 ± 10.1	0.000^b^
Encouraged by parents	53.9 ± 5.3		13.0 ± 4.3		66.9 ± 5.8	
Working with students from other departments in student council						
Yes	57.3 ± 7.1	0.008^b^	16.5 ± 6.0	0.086	73.7 ± 10.0	0.008^b^
No	55.1 ± 6.9		15.2 ± 5.6		70.3 ± 9.4	

^a^Statistically significant based on the Kruskal-Wallis test

^b^Statistically significant based on the Mann-Whitney U test

The Kruskal-Wallis statistical analysis revealed that the mean RIPLS scores differed significantly between study groups. The Mann-Whitney U statistical analysis was performed to determine significant mean differences within pairs of study groups (Table 3). The largest differences between study groups, midwifery vs nursing excepted were found in the subscale ‘professional identity and role understanding’ (*p* = 0.000). Total mean scores also differed significantly between groups (*p* = 0.000), which, however, did not also hold for the midwifery-nursing and midwifery-dentistry pairs (Table 3).

**Table 3:** Mean differences in subscales and total RIPLS scores between study groups

Study programme	Teamwork and collaboration		Professional identity and role understanding		Total RIPLS score	
	Mean	*P*	Mean	*P*	Mean	*P*
Midwifery vs Nursing	54.4 vs 54.7	0.997	10.9 vs 9.9	0.035	65.3 vs 64.7	0.349
Midwifery vs Dentistry	54.4 vs 54.9	0.867	10.9 vs 13.1	0.000*	65.3 vs 68.1	0.008
Midwifery vs Medicine	54.4 vs 56	0.037	10.9 vs 19.6	0.000*	65.3 vs 76	0.000*
Nursing vs Dentistry	54.7 vs 54.9	0.792	9.9 vs 13.1	0.000*	64.7 vs 68.1	0.001*
Nursing vs Medicine	54.7 vs 56	0.008	9.9 vs 19.6	0.000*	64.7 vs 76	0.000*
Dentistry vs Medicine	S4.9 vs 59	0.057	13.1 vs 19.6	0.000*	68.1 vs 76	0.000*

*Statistically significant based on the Mann-Whitney U test with Bonferroni correction (*p* < 0.008)

### Qualitative results: focus groups

To allow for a better interpretation of the RIPLS results, we discussed them during mono-professional focus groups. The characteristics of FG subjects were presented in Table 4. Four main themes identified, specifically: 1) Early exposure to clinical practice triggered both positive and negative perceptions of IPE and of its importance to learning communication and leadership skills, 2) Medical students caused insecurity and disengagement in other students, 3) Medical students felt pressured to be leaders, and 4) There was a need to clarify and understand each other’s profession and the boundaries of one’s own profession.

**Table 4:** Characteristics of FG participants

	Midwifery		Nurse		Dentistry		Medical	
	N	%	N	%	N	%	N	%
Gender								
Male	0	0	8	88.9	4	44.4	8	80
Female	10	100	1	11.1	5	55.6	2	20
Admission								
Invitation	1	10	1	11.1	3	33.3	1	10
Regular test	9	90	8	88.9	5	55.6	8	50
Scholarship	0	0	0	0	1	11.1	1	10
Decision to study at the programme								
Own preference	6	60	5	55.6	5	55.6	3	30
Encouraged by parents	4	40	4	44.4	4	44.4	7	70
Experiencing of working with students from different programmes								
Yes	3	30	2	22.2	4	44.4	5	50
No	7	70	7	88.8	5	55.6	5	50
	42	89.4	84	84.8	39	88.6	156	75
	Mean	SD	Mean	SD	Mean	SD	Mean	SD
Age	19.8	0.63	20.2	0.66	20.5	2.18	19.8	0.42
GPA (max score 4)	3.14	0.39	2.98	0.26	3.27	0.27	2.98	0.48

1. Early exposure to clinical practice triggered both positive and negative perceptions of IPE and of its importance to learning communication and leadership skills
  Students who favoured IPE reasoned that IPE would improve their communication skills. They believed that communication skills training during IPE would teach them to become effective communicators and improve their leadership skills:

*In my opinion*, *IPE allows us to practise our communication skills with other health professions students, so that we can identify and tackle any undesired attitudes when communicating and distributing tasks. Moreover, we doctors will be health team leaders and managers in the future; therefore we definitely have to be able to communicate well.* (Medical student)

Several nursing and midwifery students, on the other hand, indicated to have experienced unpleasant situations with medical students during early practice in hospital, causing them to be unfavourably disposed towards IPE. They explained that during clinical practice, medical students did not want to interact with them, behaved arrogantly and did not care about other health professions students. Since they felt harmonious communication between medical and nursing students in the wards was lacking, they argued that communication skills would best be taught in mono-professional courses rather than in interprofessional settings:

*At the hospital we often work mono-, rather than interprofessionally. Although we care for patients in the same ward, we never interact. We usually learn from the patient record what medical students did to the patient and what their instructions are to us; there is no face-to-face communication at all. So, we communicate via the medical record. Well, with this kind of attitude IPE will be difficult to implement* (Nursing student)

Nursing and midwifery students observed similar attitudes and significantly hierarchical behaviour among the various workers in healthcare teams during their experience in the wards:

*Once, during my internship at a hospital, I watched the nurse in charge being scolded by a specialist. The doctor was furious! The reason was basically that the nurse had not communicated a simple thing to him. We ventured to ask the nurse what it was that caused the communication problem. She explained that communicating with doctors can be difficult at times: sometimes they do not want to take phone calls, they only accept SMS, while on other days they expect the opposite. Anything would be wrong … Well, not all doctors are like this, but the phenomenon really makes us doubt whether IPE can be successful, as many doctors treat nurses unequally. The same could happen to students, right?* (Nursing student)

2. Medical students caused insecurity and disengagement in other students
  Dentistry, midwifery and nursing students had lower scores on the ‘professional identity and role understanding’ subscale of the RIPLS than medical students. The FG revealed that dentistry, midwifery and nursing students believed that mingling with students from other programmes would benefit them, although they imagined it to be difficult because medical students made them feel insecure. The widely held belief that doctors rank higher in status compared to other health professionals, together with their negative perceptions of medical students’ attitudes, caused anxiety among some nursing and midwifery students, which dented their confidence in the gains of IPE and hence mitigated their enthusiasm for IPE implementation:

*We are not as smart as medical students. Society assumes that our job is not as difficult as that of doctors, and it also puts us in position which is inferior to theirs. Medical students might consider us as their assistants. Those things are bothering me. I’m not sure if we will be able to share knowledge and views in IPE.* (Midwifery student)

3. Medical students felt pressured to be leaders
  Some medical students expressed that they were actually not too confident about studying together with students from other health programmes. They argued that IPE would better fit the clinical phase, because by then they would have sufficient medical knowledge to be able to explain their field to students from other disciplines within the IPE programme. They did not feel ready for IPE in the current pre-clinical phase, because in their view they still made too many mistakes. Such perceptions indicated that medical students felt pressured to lead and to have all the right answers, even though they were still in their pre-clinical years. As a result, some of them did not support the idea to introduce IPE:

*I don’t mean that I am against IPE, but my experience during small group discussions with fellow medical students is that sometimes we give wrong explanations with the discussion ending in deadlock because none of us know how to explain things. What will happen if students from other professions keep asking us questions which we cannot answer or to which we provide the wrong answers? The information we provide might even be misleading because our medical knowledge is not yet complete. So, I think it would be better if IPE were offered in the clinical programme.* (Medical student)

*I know that normally they* [other health professionals] *know what they have to do. But what if they have to give an injection to a patient and they ask me how many cc of the medicine is required, and I do not know either? What do you think will happen? It would be a shameful situation.* (Medical student)

4. Students felt the need to clarify and understand each other’s profession and the boundaries of one’s own profession
  Students from all programmes concurred that IPE would encourage them to better understand each other’s professional roles and responsibilities as well as the boundaries of their own roles. This became their main reason to support IPE. Students who favoured IPE also believed that IPE would improve their knowledge about medicine and clinical skills. They understood that they, as future health professionals, had professional limitations, and that, therefore, learning collaboratively with students from other health programmes would extend their knowledge and skills:

*In a variety of cases, doctors, dentists, medical specialists and nurses alike will need to collaborate. In certain dental emergency cases, for instance, we will sometimes need to refer the patient to an internal medicine specialist… so if we are offered a module that requires us to cooperate and which is meant to teach us to cooperate with other* [professionals]*, then I am in, as it will make us better practitioners in the future.* (Dentistry student)

*IPE affords us the opportunity to discuss the role of each professional. So, obviously, there will no longer be conflicts regarding roles. All health workers have nearly equal basic clinical skills. For example, both doctors and nurses can perform an infusion, give immunisation injections and so forth. However, when working together as a team it should be clear what the duties of the doctor are and what things should be done by the nurse. That can be discussed in the classroom or even before the simulation, so that we know the boundaries of each profession’s roles.* (Medical student)

A main topic arising from the FGs was the need to discuss role distribution during IPE, as unclear role boundaries were perceived to be the main source of interpersonal problems among healthcare professionals. Especially midwifery and nursing students mentioned the problem of role ambiguity (role blurring), even though some did not believe IPE could effectively address this. Nursing students, for instance, complained about role blurring in community healthcare settings, as they were sometimes forced by the community to perform medical treatments when there were no doctors available. Such activities are actually considered a violation of the law and have a negative bearing on the medical profession. Nursing students, moreover, expressed concerns that this role ambiguity in community practice would disturb IPE processes:

*We know that many doctors complain about their patients in the community being taken over by nurses. Frankly, our role in community health services is becoming blurred, because in Indonesia community nursing care is not a very popular service. As nurses can perform a number of medical treatments, it is not uncommon for them to also provide medical services. In the community, moreover, people sometimes trust a nurse more than a doctor. We are aware that it makes doctors unhappy. We fear that experiences like these will cause problems during IPE.* (Nursing student)

Likewise, some medical students voiced concerns that the fact that nurses and midwives are able to administer certain medical treatments in the community would threaten their ‘cognitive exclusivity’. More specifically, they feared that other health professions students would learn to do what was supposed to be their sole scope of practice. As a result, some of them were opposed to the IPE concept:

*I fear that, by having discussions and sharing knowledge with other professionals, students from professions other than medical, particularly nursing students, will learn more about how to handle patients, what to ask in history taking sessions and what treatment should be given. What I expect next is that, like usual, they will do it [the medical practice] themselves in their private practice in the future, although we know that they should not. I don’t want my ‘land’* [source of income] *to be taken by other professions just because of IPE.* (Medical student)

## Discussion

The present study has sought to answer the questions as to whether students in an Asian context are ready for IPE, what are the most important factors influencing students’ readiness for IPE, how students explain their readiness for IPE, and which factors either mitigate or promote this sense of readiness. To answer the first, second, and fourth question, we had the original RIPLS by Parsell and Bligh translated into Indonesian and adapted it to the Indonesian context. The translated version proved valid and reliable after an exploratory factor analysis resulting in 18 items distributed between two subscales which were renamed ‘teamwork and collaboration’ and ‘professional identity and role understanding’. The validated version differed from the original one in that the latter contained three subscales, specifically ‘teamwork and collaboration’, ‘professional identity’ and ‘roles and responsibilities’. Other studies that explored RIPLS’ validity and reliability also dismissed the third subscale [23, 24]. A recently published Indonesian version of the RIPLS reported that items 18 and 19 had low loadings, suggesting that both items did not fit the Indonesian context well [22]. The weakness of the roles and responsibilities subscale in an undergraduate setting was tentatively ascribed to respondents’ lack of professional experience [23]. This may also explain our present case, as nursing and midwifery students did have some experience of fieldwork in hospitals and public health centres, but medical and dentistry students had none. This lack of experience possibly influenced their perceptions of clear roles. Another translation of RIPLS in an Asian context with a factor solution that differed from the original version [11] was reported by Hayashi et al. [15] and exhibited high internal consistency (α = 0.87). Yet another *unadapted* Japanese version reported by Tamura et al. [25], in contrast, presented good Cronbach’s alphas for all three subscales.

Medical students’ mean scores for the RIPLS questionnaire were higher than those of students from other programmes, suggesting that they were more ready for IPE compared to the other three groups. At the same time, one could infer that the fact that medical students had not previously been exposed to clinical practice allowed them to remain idealistic. Contrary to our finding, other studies have found that the mean RIPLS scores of medical students were actually lower than those of students from other health professions programmes [26, 27]. Additionally, nursing students have been reported to be more receptive to the idea of collaborating with other health professionals compared to medical students [12].

We also found that the RIPLS score seemed to correlate with the study programme chosen, which was especially true for the ‘professional identity and role understanding’ subscale. This finding appears to confirm previous research which has suggested that students’ attitudes towards IPE differ according to their professional background [28]. Similarly, we found a correlation between students’ GPA and their perceptions of IPE. Again, this finding is consistent with previous research demonstrating that students with a high cognitive capacity seem to be more ready to learn with students from other disciplines in IPE [18]. Intrinsic motivation to study in a health professions programme was also found to affect students’ perceptions of teamwork and collaboration with other health professionals, as well as of interprofessional education.

Students who had already collaborated with colleagues from other departments in the student council had a more positive attitude towards teamwork and collaboration, as well as towards interprofessional education in general. Such opportunities to interact and learn together with other professionals nurture the development of communication skills [19], leadership skills and collaborative skills [29]. Hence, involvement in multi-professional student activities will increase students’ readiness for IPE.

To complement findings from the RIPLS questionnaire and to specifically address the third research question of how students explain their readiness for IPE, we conducted four FGDs. From these discussions it resulted that medical students’ good performance on the RIPLS questionnaire was not reflected in feedback received from nursing and midwifery students, who had already been exposed to collaborative clinical care. Instead, they reported that communication with medical students during their clinical exposure was minimal. In their perception, medical students were unwilling to communicate, behaved arrogantly and held stereotyped views, attributes which inhibit the implementation of IPE [9, 30]. In addition, clinical education was organised by each health professions programme separately, with distinct learning schedules, activities and assessment procedures, and organisers did not communicate with each other. As a result, students rarely interacted with students from other programmes, not even when treating a patient together. The fact that several students had been active in the student council did not play a role in this, as the context is entirely different.

Although some students believed that IPE would improve the quality of collaboration within a team of health professionals, early exposure to professional practice could also cause students to have negative perceptions of the healthcare team as well as of IPE. Nursing and midwifery students, for instance, had experienced that interactions within healthcare teams in hospitals were not always harmonious, which, in turn, seemed to kindle negative perceptions of collaborative interprofessional healthcare teams, as well as of other health professionals. Since medical and dentistry students had not had any practical experience in hospitals, they may have held idealistic views about improving communication and leadership skills through an IPE programme. It has been reported in the literature that students generally learn their discipline’s attitudes, norms, values and practices through tacit observation of staff behaviours [31, 32]. When what they see and learn in real practice are discipline-bound stereotypes and communication problems, this might seriously interfere with the development of collaborative practice. Consequently, students may develop negative opinions about interprofessional interaction [33] and negative perceptions of the importance of IPE. In a similar vein, Makino et al. [14] examined the relationship between exposure to clinical practice and attitudes towards interprofessional healthcare teams using the modified Attitudes towards Healthcare Teams Scale (ATHCTS). He found that alumni had significantly lower overall mean scores than undergraduate students, inferring that exposure to clinical practice may detract from the positive attitudes students have towards the efficacy of healthcare teams. Underlying issues reported as barriers to collaboration were the fact that fresh graduates were often ill-prepared to apply their knowledge to real-world problems [34] and to cope with the competitive spirit dominating the workplace [35], which in turn engendered negative attitudes towards interprofessional care and learning. This may also explain why the midwifery and nursing students of our study who had already been exposed to clinical practice were less favourable to IPE compared to the medical and dentistry students who had no prior clinical experience. This finding reinforces how students learn from role modelling [36], making it imperative that healthcare team interactions in all healthcare settings be improved. We need to find well-functioning healthcare teams that can serve as role models for students so that they can learn how to effectively communicate with other health professionals during patient care.

Another finding identified from the FGDs is that medical students caused insecurity and disengagement in other students indicating that deeply ingrained societal views permeate students’ perceptions of IPE. The view that other health professionals would be inferior to doctors caused nursing and midwifery students to be insecure about IPE. They felt that they ranked lower in academic status and that their intended profession was less ‘prestigious’ than medicine. It has indeed been reported that nursing students were perceived inferior to medical students with respect to several characteristics, including status in society, competence and academic ability [37]. As a result, students developed stereotypical notions of how other health professions students would behave towards them during interprofessional learning [38], which ideas, in turn, dented their confidence about learning collaboratively with medical students in IPE. A number of medical students also exhibited little confidence in their own performance, knowledge and capacity to be leaders of healthcare teams. When it comes to leadership skills, we have learned from the literature that, although students perceive themselves as competent communicators, they also consider themselves less effective care managers [39]. For this reason, efforts to implement IPE have specifically aimed at the incorporation of experiential leadership training [40].

Students appreciated the fact that unclear boundaries between health professionals’ roles complicate interprofessional collaboration in Indonesia, making it an important issue to address during IPE. However, unclear role boundaries and role blurring were additional reasons for some students to have negative perceptions of IPE. This effect has been reported elsewhere in the literature and is considered to be a problem among healthcare professionals [41]. Medical students, for instance, opposed the concept of IPE since they did not want to share knowledge with other health professionals. With this attitude students sought to defend their ‘cognitive exclusivity’ against current healthcare practices in Indonesia which allow nurses to offer private medical services, providing therapeutic treatment to patients, prescribing medicines, and other tasks that actually pertain to the doctor’s scope of practice. Sometimes, this is partially the result of doctors transferring their practices to the larger towns, leaving the care of patients in villages to nurses. Additionally, it is not uncommon for nurses to deliver medical services at a lower rate to people in the lower echelons, so that those who suffer most from disease are often treated by nurses rather than doctors [42]. Such role conflicts among health professionals, especially between nurses and doctors, are commonplace in healthcare services [43]. This issue is particularly sensitive in Indonesia and perhaps in other Asian countries with similar backgrounds and caused students to be reluctant to share knowledge within IPE.

This research contribute to literature as, to the best of our knowledge, it was the only study that examined students’ perception toward interprofessional education applying mixed method design, which allows it to explore comprehensive information of students’ perception. Other studies on the same theme generally applied quantitative design. However, there is limitation of this study that the students in each group were not equal in number, which likely to cause bias. To minimise the bias, data were taken from entire accessible population and the statistical calculation of quantitative data were based on average values. In addition, data were collected from schools of health profession of a university in Indonesia, which might not represent all Indonesian students. The findings might be difficult to generalise as the data were taken from one institution only. Similar study could be conducted with broader population.

## Conclusion

Medical students’ mean scores for the RIPLS questionnaire were higher than those of students from other programmes. The study programme chosen, GPA, intrinsic motivation and experience of working with students from other study programmes in a student council were factors that influenced perceptions of IPE. Some themes that identified during the focus groups were: early exposure to clinical practice triggered both positive and negative perceptions of IPE and of its importance to learning communication and leadership skills; medical students caused insecurity and disengagement in other students; medical students felt pressured to be leaders; and there was a need to clarify and understand each other’s profession and the boundaries of one’s own profession. In order for IPE to be successful in the Asian context and culture, heed should be paid to the blurring of roles and role boundaries. We need strong role models from the various health professions to help create and implement successful IPE programmes and ultimately improve interprofessional collaboration in the Indonesian healthcare system.

## Abbreviations

ATHCTS, Attitudes Towards Healthcare Teams Scale; FG, Focus Group; FGD, Focus Group Discussion; GPA, Grade Point Average; IPE, Interprofessional education; KMO, Kaiser-Meyer-Olkin; RIPLS, Readiness for Interprofessional Learning Scale.

## Additional files

Additional file 1: RIPLS Validation. (DOCX 19 kb) Additional file 2: Contribution of components to each subscale. (DOCX 17 kb)

## References

[1] Besner J (2008). Interprofessional practice rhetoric or reality?. The Canadian Nurse.

[2] Jacobsen F, Lindqvist S (2009). A two-week stay in an interprofessional training unit changes students’ attitudes to health professionals. Journal Of Interprofessional Care.

[3] Cragg B, Hirsh M, Jelley W, Barnes P (2010). An interprofessional rural clinical placement pilot project. Journal Of Interprofessional Care.

[4] Kenaszchuk C, MacMillan K, VanSoeren K, Reeves S (2011). Interprofessional simulated learning: short-term associations between simulation and interprofessional collaboration. BMC Medicine.

[5] Maeno T, Takayashiki A, Anme T, Tohno E, Maeno T, Hara A (2013). Japanese students’ perception of their learning from an interprofessional education program: a qualitative study. International Journal of Medical Education.

[6] Houben V (2014). Socio cultures of insular southeast Asia: between history, area and social studies. Transcience.

[7] Sujatmiko G (2011). Social exclusion and Inclusion policy in Indonesia. Int J Bus Soc Sci.

[8] Chongsuvivatwong V, Phua KH, Yap MT (2011). Health and health-care systems in Southeast Asia: diversity and transitions. Lancet.

[9] Oandasan I, Reeves S (2005). Key elements of interprofessional education. Part 2: factors, processes and outcomes. J Interpr of Care.

[10] Barret M, Carolyn G, Christine A, Reena A, Molly R (2013). Dissecting first-year students’ perceptions of health profession groups: potential barriers to interprofessional education. Journal Of Allied Health.

[11] Parsel G, Bligh J (1999). The development of a questionnaire to assess the readiness of health care students for interprofessional learning (RIPLS). Med Educ.

[12] Horsburgh M, Lamdin R, Williamson E (2001). Multiprofessional learning: the attitudes of medical, nursing and pharmacy students to shared learning. Med Educ.

[13] Jacqui C, Mingsheng L (2008). Asian students’ voices: an empirical study of Asian students’ learning experiences at a New Zealand University. J Stud Int Educ.

[14] Makino T, Shinozaki H, Hayashi K, Lee B, Matsui H, Kururi N, Kazama H, Ogawara H, Tozato F, Iwasaki F, Asakawa Y, Abe Y, Uchida Y, Kanaizumi S, Sakou K, Watanabe H (2013). Attitudes toward interprofessional healthcare teams: a comparison between undergraduate students and alumni. Journal Of Interprofessional Care.

[15] Hayashi T, Shinozaki H, Makino T, Ogawara H, Asakawa H, Iwasaki K, Matsuda T, Abe Y, Tozato F, Koizumi M, Yasukawa T, Lee B, Hayashi K, Watanabe H (2012). Changes in attitudes toward interprofessional health care teams and education in the first- and third-year undergraduate students. Journal Of Interprofessional Care.

[16] Johnson RB, Onwuegbuzie AJ (2004). Mixed methods research: a research paradigm whose time has come. Educ Res.

[17] Giordano C, Dissecting First Year Students’ Perception on Health Profession Groups (2013). Potential Barrier to Interprofessional Education. J Allied Health.

[18] Sargeant J, Loney E, Murphy G (2008). Effective interprofessional teams: “contact is not enough” to build a team. J Contin Educ Health Prof.

[19] Morisson S, Jenkins J (2007). Sustained effects of interprofessional shared learning on student attitudes to communication and team working depend on shared learning opportunities on clinical placement as well as in the classroom. Medical Teacher.

[20] Mega C, Ronconi L, DeBeni R (2014). What makes a good student? How emotions, self-regulated learning, and motivation contribute to academic achievement. J Educ Psychol.

[21] Sarantakos S (2012). Social Research 4th edition.

[22] Tyastuti D, Onishi H, Ekayanti F, Kitamur K (2014). Psychometric item analysis and validation of the Indonesian version of the Readiness for Interprofessional Learning Scale (RIPLS). J Interprof Care.

[23] McFadyen A, Webster V, Strachan K, Figgins E, Brown H, McKechnie J (2005). The Readiness for Interprofessional Learning Scale: a possible more stable sub-scale model for the original version of RIPLS. Journal Of Interprofessional Care.

[24] Mahler C, Rochon J, Karstens S, Szecsenyi J, Hermann K (2014). Internal consistency of the readiness for interprofessional learning scale in German health care students and professionals. BMC Medical Education.

[25] Tamura Y, Seki K, Usami M, Taku S, Bontje P, Ando H, Taru C, Ishikawa Y (2012). Cultural adaptation and validating a Japanese version of the readiness for interprofessional learning scale (RIPLS). Journal Of Interprofessional Care.

[26] Reid R, Bruce D, Allstaff K, McLernon D (2006). Validating the Readiness for Interprofessional Learning Scale (RIPLS) in the postgraduate context: are health care professionals ready for IPL?. Med Educ.

[27] El-Zubeir M, Rizk D, Al-Khali R (2006). Are senior UAE medical and nursing students ready for interprofessional learning? Validating the RIPL scale in a Middle Eastern context. J Interprof Care.

[28] Curran VR, Sharpe D, Flynn K, Button P (2010). A longitudinal study of the effect of an interprofessional education curriculum on student satisfaction and attitudes towards interprofessional teamwork and education. J Interprof Care.

[29] Hoffman SJ, Rosenfield D, Gilbert JH, Oandasan IF (2008). Student leadership in interprofessional education: benefits, challenges and implications for educators, researchers and policymakers. Med Educ.

[30] Tunstall-Pedoe S, Rink E, Hilton S (2003). Student attitudes to undergraduate interprofessional education. J Interpr of Care.

[31] Lave J, Wenger E (2003). Situated Learning Legitimate Peripheral Participation.

[32] Russell L, Nyhof-Young J, Abosh B, Robinson S (2006). An exploratory analysis of an interprofessional learning environment in two hospital clinical teaching units. J Interprof Care.

[33] Pollard K, Miers M, Gilchrist M (2004). Collaborative learning for collaborative working? Initial findings from a longitudinal study of health and social care students. Health and Social Care in the Community.

[34] Blouin R, Joyner P, Pollack G (2008). Preparing for a renaissance in pharmacy education: the need, opportunity, and capacity for change. American Journal Pharmacy Education.

[35] Tremblay D, Drouin D, Lang A, Roberge D, Ritchie J, Plante A (2010). Interprofessional collaborative practice within cancer teams: translating evidence into action. A mixed methods study protocol. Implement Sci.

[36] Selle KM, Salamon K, Boarman R, Sauer J (2008). Providing interprofessional learning through interdisciplinary collaboration: the role of “modelling”. Journal Of Interprofessional Care.

[37] Rudland JR, Mires GJ (2005). Characteristics of doctors and nurses as perceived by students entering medical school: implication for shared teaching. Med Educ.

[38] Reeves S (2000). Community-based interprofessional education for medical, nursing and dental students. Health Soc Care Community.

[39] Varkey P, Peloquin J, Reed D, Lindor K, Harris I (2009). Leadership curriculum in undergraduate medical education: a study of student and faculty perspectives. Med Teach.

[40] Eubank D, Geffken D, Orzano J, Ricci R (2012). Teaching adaptive leadership to family medicine residents: What? Why? How?. Families, Systems, & Health.

[41] Guru R, Siddiqui MA, Ur-Rehman A (2013). Professional identity (Role Blurring) of occupational therapy in community mental health in India. Isra Medical Journal.

[42] Sciortino RME (1992). Care-takers of cure. A study of health centre nurses in rural Central Java.

[43] Brown J, Lewis L, Ellis K, Stewart M, Freeman TR, Kasperski MJ (2011). Conflict on interprofessional primary health care teams – can it be resolved?. Journal Of Interprofessional Care.

