# Supplementary material for: Understanding students’ readiness for interprofessional learning in an Asian context: a mixed-methods study
Source: BMC Med Educ. 2016 Jul 15;16:179. doi: 10.1186/s12909-016-0704-3 (PMC4946087; doi:10.1186/s12909-016-0704-3)
Supplement: Additional file 1: — RIPLS Validation. (DOCX 19 kb) [file 12909_2016_704_MOESM1_ESM.docx]

**Additional file 1: RIPLS Validation**

KMO > 0.5 indicates that the results of factorial analysis can be applied.
