# Supplementary material for: Understanding students’ readiness for interprofessional learning in an Asian context: a mixed-methods study
Source: BMC Med Educ. 2016 Jul 15;16:179. doi: 10.1186/s12909-016-0704-3 (PMC4946087; doi:10.1186/s12909-016-0704-3)
Supplement: Additional file 2: — Contribution of components to each subscale. (DOCX 17 kb) [file 12909_2016_704_MOESM2_ESM.docx]

**Additional file 2: Contribution of components to each subscale**

| **No** | **Statements** | teamwork and collaboration | Professional identity and role understanding | Q19 |
| --- | --- | --- | --- | --- |
| 1 | *Learning with other students will help me become a more effective member of a healthcare team.* | 0.767 |  |  |
| 2 | *Patients would ultimately benefit if healthcare students worked together to solve patient problems.* | 0.805 |  |  |
| 3 | *Shared learning with other healthcare students will increase my ability to understand clinical problems* | 0.830 |  |  |
| 4 | *Learning with heath care students before qualification would improve relationships after qualification* | 0.799 |  |  |
| 5 | *Communication skill should be learnt with other healthcare students* | 0.796 |  |  |
| 6 | *Shared learning will help me to think positively about other healthcare students* | 0.853 |  |  |
| 7 | *For small group learning to work, students need to trust and respect each other* | 0.767 |  |  |
| 8 | *Team-working skills are essential for all healthcare students to learn* | 0.772 |  |  |
| 9 | *Shared learning will help me to understand my own limitations* | 0.801 |  |  |
| 10 | *I don’t want to waste my time learning with other healthcare students ^a^* |  | 0.919 |  |
| 11 | *It is not necessary for undergraduate healthcare students to learn together ^a^* |  | 0.922 |  |
| 12 | *Clinical problem-solving skills can only be learnt with students from my own department ^a^* |  | 0.876 |  |
| 13 | *Shared learning with other healthcare students will help me to communicate better with patients and other professionals* | 0.692 |  |  |
| 14 | *I would welcome the opportunity to work on small group projects with other healthcare students* | 0.741 |  |  |
| 15 | *Shared learning will help me to clarify the nature of patient problems* | 0.765 |  |  |
| 16 | *Shared learning before qualification will help me become a better team worker* | 0.688 |  |  |
| 17 | *The purpose of nurses and therapists is mainly to provide support for doctors* |  | 0.678 |  |
| 18 | *I’m not sure what my professional role will be* |  | 0.825 |  |
| 19 | *I have to acquire much more knowledge and skills than other healthcare students* |  |  | 0.741 |

We used Parsell and Bligh’s RIPLS (1999) as instrument; negatively worded items were reverse-scored.
